# Nutrition in Pediatric Patients and Vulnerable Populations: Updates and Advances

**DOI:** 10.3390/children11040430

**Published:** 2024-04-03

**Authors:** Maria G. Grammatikopoulou, Tonia Vassilakou

**Affiliations:** 1 Immunonutrition and Clinical Nutrition Unit, Department of Rheumatology and Clinical Immunology, Medical School, University of Thessaly, Biopolis Campus, GR-43100 Larissa, Greece; 2 Department of Public Health Policy, School of Public Health, University of West Attica, GR-11521 Athens, Greece

## 1. Introduction

Nutrition is a modifiable factor of paramount importance for the prevention and attainment of health and the development of youngsters. Improved nutritional status and diets of adequate quality are associated with better infant, child, and adolescent health, improved growth and development, stronger immune systems, a lower risk of non-communicable diseases (NCDs), and longevity [1,2,3,4]. These associations become more profound when vulnerable populations are concerned [5,6]. In this Special Issue of *Children,* we explore the role of nutrition in pediatric patients and vulnerable children, presenting recent updates and advances regarding the nutritional status and medical nutrition therapy of these populations.

## 2. Burden of Malnutrition and Food Insecurity

The burden of malnutrition remains a worldwide epidemic, with specific geographic areas accounting for the greatest burden globally [7]. Since the Convention on the Rights of the Child (1989) and the joint consortium held by the World Health Organization (WHO) and the United Nations Children’s Fund (UNICEF) leading to the “Ten Steps to Successful Breastfeeding”, many policymakers and scientific societies have produced reports on the effective promotion of healthy nutrition among youngsters (contribution 1). With this in mind, Sotiraki et al. (contribution 1) provided a comprehensive critical discussion of such policies and action plans aiming to promote healthy nutrition among infants and children globally. These include the management of acute malnutrition, the prevention of childhood overweight and obesity, and setting healthy nutrition as a policy goal, including the promotion of breastfeeding.

In the same vein, Charle-Cuéllar et al. used a case-control design to evaluate the effectiveness and coverage of treatment for severe acute malnutrition (SAM) in the Guidimakha Region, Mauritania (contribution 2). They argued that the integration of SAM treatment as part of the package delivered by community health workers (CHWs) in Mauritania may improve the prompt identification of malnutrition cases and the improved access to treatment services and, by inference, ameliorate SAM’s clinical outcomes (contribution 2). After all, according to UNICEF and the Global Nutrition Cluster [8,9], countries ought to adopt different modalities of simplified approaches to increase coverage of acute malnutrition, including the decentralization of SAM treatment using CHWs for its delivery. Their results showed no difference in the proportion of children cured from SAM between the intervention and control arms. However, in the intervention zone, coverage for SAM increased from 53.6% to 71.7%, whereas it remained at similar levels (44%) in the control zone. The results indicate that the decentralization model of community health workers treating SAM in Mauritania improves SAM treatment coverage and complies with the international quality standards for treating SAM in a community setting (contribution 2).

Shafiq and colleagues (contribution 3) assessed gender disparity in the prevalence of malnutrition (girls malnourished in households/boys malnourished in households) in Pakistan, using data from the Pakistan Demographic and Health Survey (PDHS). The results revealed that households with good socioeconomic status—those with more household members, a greater maternal educational level, maternal employment, and the male head of the household being present—reduced gender disparity in the nutrition intake of children (contribution 3).

Finally, Aggeli et al. (contribution 4) evaluated food insecurity (FI) in pairs of mothers and their children in Greece. The results revealed that approximately ¼ of the pairs experienced some degree of FI, with a higher prevalence (64.7%) among single-mother families (contribution 4). Lower FI was apparent in households with higher maternal educational attainment and a conjugal family status (contribution 4). Maternal adherence to the traditional Mediterranean diet (MD) was inversely related to the respective adherence of their offspring, suggesting that during periods of financial constraints, maternal diet quality is compromised at the expense of affording a better diet for the minors in the family (contribution 4). The results indicate the need for early identification of FI, especially among single parent families with the mother as the primary caregiver, suggesting an increased vulnerability to FI.

## 3. Milk, Please!

With breastfeeding being a priority in tackling global malnutrition in infants (contribution 1), according to UNICEF [10], the private sector can also support this goal by helping mothers breastfeed after birth and within the workplace through supportive parental and maternal leave policies, supportive workplace policies, and the provision of sufficient time and appropriate spaces for breastfeeding or expressing and storing breastmilk. Recent research conducted in Lebanon (contribution 5) revealed that maternal occupation is inversely associated with breastfeeding practices. Although supporting breastfeeding in the hospital setting and husband’s support were related to increased breastfeeding odds, male offspring, maternal coronavirus disease 2019 (COVID-19) diagnosis, and bottle feeding at the hospital were factors associated with the early initiation of solid foods at 4 months of age (contribution 5).

The key points of this study are numerous. First and foremost, it appears that maternal employment remains a barrier to breastfeeding for many mothers globally, as seen in the USA [11], China [12], and many more countries, and returning to the workplace continues to remain one of the most common reasons for terminating breastfeeding all over the world [13]. This indicates that breastfeeding-friendly employment strategies are not available yet for all, forcing a great percentage of mothers to introduce solid foods early in order to “balance” infant nutrition as breastfeeding is limited due to economic and employment reasons.

The second takeaway message of this study involves the relationship between COVID-19 diagnosis and infant bottle feeding. When a public health emergency was declared for COVID-19 and research on the virus was growing exponentially [14], Chinese experts [15] contraindicated breastfeeding practices in cases of infected mothers, based on their knowledge stemming from SARS (Severe Acute Respiratory Syndrome), while taking into account the possible SARS-CoV-2 transmission. Since then, however, research has shown that samples of breast milk from suspected or infected mothers mainly tested negative in SARS-CoV-2 viral tests, whereas the majority of infants who were breastfed by COVID-19-positive mothers were also negative for SARS-CoV-2 [16]. In Layman’s terms and based on van de Perre’s criteria [17], the chances of SARS-CoV-2 being transmitted vertically through breast milk were minimal [16]. Based on the available data, several researchers and authorities continued to promote breastfeeding, even among infected mothers [18,19,20]. However, it appears that in many cases, fear of transmission resulted in the discontinuation of breastfeeding, as seen in Lebanon (contribution 5). In parallel, according to Latorre [21], early discharge from the hospital due to lockdown restrictions and safety measures induced an additional decrease in exclusively breastfeeding even among non-infected mothers.

## 4. Body Weight Perception and Body Weight Status

Overweight and obesity remain an important issue of concern among all age groups and in particular among children and adolescents [22], indicating the lack of political will and implementation of obesity-prevention strategies [23]. Research suggests that girls are more sensitive regarding their weight status, with a great percentage being dissatisfied with their body image [24]. In this issue, Kanellopoulou and associates (contribution 6) revealed that children who follow a healthy dietary pattern also tend to have a more accurate weight perception, one in accordance with their actual weight, and lower odds of being overweight/obese. Similar findings were also reported by Kontele et al. (contribution 7) in a sample of adolescent female gymnasts, with greater adherence to the MD and diet quality being related to a healthier body mass index (BMI). It all points to the fact that diet quality and body weight status are interrelated.

Additionally, Jančič and colleagues (contribution 8) evaluated the relationship between body composition indices and the health status of children and adolescents using bioelectrical impedence analysis (BIA). The results revealed a significant association between body composition parameters and a variety of health indices, including systolic blood pressure, fasting plasma insulin levels, serum creatinine, urate, liver function proxies, total cholesterol, triglycerides (TG) and apolipoproteins, homocysteine, vitamin D concentrations, as well as urine protein levels (contribution 8). In parallel, gender differences in body composition were observed between participants with and without hepatic steatosis, as well as among those with and without left ventricular hypertrophy (contribution 8). Subsequently, it appears that obesity and body composition are important contributors to the development of cardiovascular risk factors and do not appear to be dependent solely on fat mass. Thus, body composition can be used as a proxy for determining cardiovascular risk.

## 5. Feeding and Eating Disorders (FEDs)

Feeding and eating disorders (FEDs) are important effectors of children and adolescents’ health status, with an increasing prevalence during the past few years [25]. Female sex, increasing age, and BMI are factors associated with greater FED practices among children and adolescents [25]. Often, FEDs, Other Specified FEDs (OSFEDs), and Unspecified FEDs (USFEDs) accompany a chronic disease diagnosis where body weight is affected by medication [26,27] or where nutrition is an integral therapeutic component in disease self-management [28].

In the present issue, Petropoulou and associates (contribution 9) reviewed the literature on the prevalence of FEDs and disordered eating practices among children and adolescents with cystic fibrosis (CF). Although children with CF are prone to malnutrition and are mainly underweight or of normal body weight [29,30,31], research also indicates that those who struggle with body weight issues tend to follow disordered eating practices (contribution 9). Visible scars, ports, or tubes and the need for supplementary oxygen supply appear to influence body image perception among youngsters with CF, propelling disordered eating practices (contribution 9).

Additionally, Tsakona and associates (contribution 10) presented the case of an adolescent girl with type 1 diabetes mellitus (T1DM), anxiety disorder, and anorexia nervosa (AN). Their therapeutic approach included dietary counseling, physiotherapeutic relaxation sessions, and breathing exercises for 3 weeks, which resulted in stress relief, an increased BMI, subsided AN symptoms, and the achievement of a “normal” menstrual cycle (contribution 10).

## 6. Food Allergies (FAs)

IgE-mediated food allergies (FAs) have been shown to affect more than 10% of the US population, whereas more individuals also suffer from FAs without convincingly IgE-mediated symptoms [32]. Although the burden of FAs is highly heterogeneous [33], the condition affects all age groups but pediatric populations in a higher degree, all races, and people from every socioeconomic strata, greatly affecting food choices and dietary behaviors while carrying significant economic treatment costs [34].

Drakouli et al. (contribution 11) systematically reviewed all primary research on the quality of life (QoL) among children with FAs. Few studies compared QoL between children with FAs and healthy children, with three of them showing worse QoL among the first compared to the latter. Immunotherapy improved the QoL of children with FAs; however, history of anaphylaxis, multiple FAs, additional allergies, and the number and severity of symptoms were the main factors associated with a reduced QoL (contribution 11).

## 7. Juvenile Idiopathic Arthritis (JIA)

Juvenile idiopathic arthritis (JIA) involves a complex variety of heterogenous arthritides with an onset prior to 16 years of age [35], consisting of the most frequent chronic, autoimmune, rheumatic, and musculoskeletal (RMD) diseases encountered during childhood [36]. In this issue, the nutritional aspects of JIA are detailed (contribution 12), providing a comprehensive guide for dietitians. From treatment side effects, micronutrient deficiencies, delayed growth, and overweight to oral and gastrointestinal problems limiting dietary intake, all available evidence is pooled herein for health professionals involved in JIA treatment. This review also highlights the importance of registered dietitians and nutritionists (RDNs) in JIA management, calling for more specialized training among RDNs and pediatric rheumatologists (contribution 12).

## 8. Familial Chylomicronemia Syndrome (FCS)

Familial chylomicronemia syndrome (FCS) is a rare autosomal recessive disorder characterized by extremely high TG levels and severe clinical symptoms, including recurrent acute pancreatitis, abdominal pain, and hepatosplenomegaly, all associated with a worse prognosis and higher mortality rates [37]. The first line of treatment for this condition is dietary, consisting of a lifelong, very strict low-fat diet that aims to do the following: (1) limit fat to <15–20 g/day (often corresponding to <10–15% of the total daily energy intake when adults are concerned); (2) meet the recommendations for essential fatty acids (α-linolenic and linoleic); (3) supplement orally with fat-soluble vitamins, minerals, and medium-chain triglyceride (MCT) oil to meet recommendations; (4) consume complex carbohydrate foods instead of simple or refined carbohydrate sources; and (5) adjust energy intake for managing body weight accordingly [38]. With this strict dietary treatment, adherence to the dietary recommendations is challenging, especially for pediatric patients.

In this issue, Thajer and associates (contribution 13) presented a follow-up study of a small pediatric FCS cohort consisting of four patients. The results showed that, as far as children with FCS are concerned, the recommendation regarding the reduction in fat intake to <10–15% of the total daily energy intake is not feasible, prompting a readjustment of the guidelines (contribution 13).

Nonetheless, dietary fat restriction between 10–26% of the total daily energy intake appears to be both feasible and effective for treating genetically confirmed FCS in children. In parallel, the pediatric FCS traffic light tool and the age-appropriate portion sizes for pediatric patients with FCS can aid children and their families in improving adherence to the strict low-fat diet for life (contribution 13).

## 9. Are Guidelines Fit-for-Purpose?

Following the clinical practice guidelines (CPGs) is the mainstay of evidence-based practice; however, how fit-for-purpose are the guidelines we follow? In a comprehensive review, all CPGs for the nutritional management of CF were evaluated (contribution 14), using the Appraisal of Guidelines, Research, and Evaluation II (AGREE II) instrument and checklist [39]. This included a total of 22 CPGs (seven solely nutrition-oriented) by 14 distinct publishers. The results indicated that not all CPGs follow state-of-the-art standards of development. Among those evaluated, the Thoracic Society of Australia and New Zealand CPGs [40] scored the highest overall quality, while the Paediatric Gastroenterology Society/Dietitians Association of Australia CPGs [41] demonstrated the lowest quality score. The study indicates the need for the development of more robust, unbiased, state-of-the-art CPGs to aid everyday clinical practice.

## 10. Conclusions

The present issue adds valuable knowledge to disease-specific dietary conditions in children as well as to the nutritional issues of vulnerable children. For this, we hope it is useful and practical for those working with pediatric populations and vulnerable children.
